# Correlating Sensory Assessment of Smoke-Tainted Wines with Inter-Laboratory Study Consensus Values for Volatile Phenols

**DOI:** 10.3390/molecules27154892

**Published:** 2022-07-30

**Authors:** James W. Favell, Kerry L. Wilkinson, Ieva Zigg, Sarah M. Lyons, Renata Ristic, Carolyn J. Puglisi, Eric Wilkes, Randell Taylor, Duane Kelly, Greg Howell, Marianne McKay, Lucky Mokwena, Tim Plozza, Pei Zhang, AnhDuyen Bui, Ian Porter, Orrin Frederick, Jasha Karasek, Colleen Szeto, Bruce S. Pan, Steve Tallman, Beth Anne McClure, Hui Feng, Eric Hervé, Anita Oberholster, Wesley F. Zandberg, Matthew Noestheden

**Affiliations:** 1 Department of Chemistry, The University of British Columbia, Kelowna, BC V1V 1V7, Canada; jfavell@ualberta.ca (J.W.F.); ieva.zigg@ubc.ca (I.Z.); sarah@suprarnd.ca (S.M.L.); 2 Department of Wine Science, Waite Research Institute, The University of Adelaide, PMB 1, Glen Osmond, SA 5064, Australia; kerry.wilkinson@adelaide.edu.au (K.L.W.); renata.ristic@adelaide.edu.au (R.R.); carolyn.puglisi@adelaide.edu.au (C.J.P.); colleen.szeto@adelaide.edu.au (C.S.); 3 The Australian Research Council Training Centre for Innovative Wine Production, Glen Osmond, SA 5064, Australia; 4 Supra Research and Development, Kelowna, BC V1X 6Y5, Canada; 5 The Australian Wine Research Institute, P.O. Box 197, Glen Osmond, SA 5064, Australia; eric.wilkes@awri.com.au (E.W.); randell.taylor@awri.com.au (R.T.); 6 Vintessential Laboratories, Dromana, VIC 3936, Australia; duanekelly@hotmail.com (D.K.); greg@ferment.com.au (G.H.); 7 Department of Viticulture and Oenology/Central Analytical Facility, Stellenbosch University, Stellenbosch 7600, South Africa; marianne@sun.ac.za (M.M.); mokwenal@sun.ac.za (L.M.); 8 Agriculture Victoria, Bundoora, VIC 3083, Australia; tim.plozza@agriculture.vic.gov.au (T.P.); p.zhang@latrobe.edu.au (P.Z.); anhduyen.bui@agriculture.vic.gov.au (A.B.); 9 School of Life Sciences, LaTrobe University, Bundoora, VIC 3083, Australia; i.porter@latrobe.edu.au; 10 Enartis Vinquiry, Windsor, CA 95492, USA; orrin.frederick@enartis.com (O.F.); jasha.karasek@enartis.com (J.K.); 11 E. & J. Gallo Winery, Modesto, CA 95354, USA; bruce.pan@ejgallo.com (B.S.P.); steve.tallman@ejgallo.com (S.T.); bethanne.mcclure@ejgallo.com (B.A.M.); hui.feng@ejgallo.com (H.F.); 12 ETS Laboratories, St Helena, CA 94574, USA; eherve@etslabs.com; 13 Department of Viticulture and Enology, University of California Davis, Davis, CA 95616, USA; aoberholster@ucdavis.edu

**Keywords:** fire, grapes, inter-laboratory study, gas chromatography-mass spectrometry, rate-all-that-apply, smoke, smoke taint, volatile phenols, wine

## Abstract

Vineyard exposure to wildfire smoke can taint grapes and wine. To understand the impact of this taint, it is imperative that the analytical methods used are accurate and precise. This study compared the variance across nine commercial and research laboratories following quantitative analysis of the same set of smoke-tainted wines. In parallel, correlations between the interlaboratory consensus values for smoke-taint markers and sensory analyses of the same smoke-tainted wines were evaluated. For free guaiacol, the mean accuracy was 94 ± 11% in model wine, while the free cresols and 4-methylguaiacol showed a negative bias and/or decreased precision relative to guaiacol. Similar trends were observed in smoke-tainted wines, with the cresols and glycosidically bound markers demonstrating high variance. Collectively, the interlaboratory results show that data from a single laboratory can be used quantitatively to understand smoke-taint. Results from different laboratories, however, should not be directly compared due to the high variance between study participants. Correlations between consensus compositional data and sensory evaluations suggest the risk of perceivable smoke-taint can be predicted from free cresol concentrations, overcoming limitations associated with the occurrence of some volatile phenols, guaiacol in particular, as natural constituents of some grape cultivars and of the oak used for barrel maturation.

## 1. Introduction

The flavor profile of a wine is heavily dependent on its chemical composition [1], which is influenced, in part, by a large number of viticultural factors [2]. Unwanted alterations in grape/wine chemistry can result in faults in a wine’s sensory profile and potentially affect its marketability [3,4]. Smoke-taint is an increasingly common fault that may occur in wines made from grapes that have been exposed to smoke from wildfires during key phenological stages [5,6]. Smoke-tainted wines are characterized by smoky aromas and flavors and may additionally possess an ashy aftertaste [7]. Climate change models predict an increase in the severity of worldwide wildfire seasons over the coming years [8,9], and thus, it is expected that the threat smoke-taint poses to the global wine-making industry will be recurrent and increasingly problematic. Indeed, the impact of changing wildfire patterns on the wine industry has been keenly felt by the western American/Canadian, Australian, South African, and Chilean grape-growing regions over the last five years. In these regions, severe wildfire events have resulted in hundreds of millions of dollars of lost revenue due not only to physical damage to vines and infrastructure but also to the presence of smoke-taint in grapes. For example, in 2020, wildfires in parts of Australia led to an estimated AUD 400 million loss with an AUD 140 million loss in wine revenue from several regions in North-East Victoria [10], while the California wine industry saw over USD 400 million in losses in the same year due to a combination of property, crop and wine losses [11].

There is a strong correlation between smoke-taint and elevated concentrations of free volatile phenols (VPs) in wine grapes [12,13]. VPs can be introduced to wine: (1) as a by-product of oak exposure during winemaking; (2) due to the presence of spoilage organisms; or (3) via endogenous biosynthesis in planta [14,15,16,17,18]. Most relevantly though, VPs present in wildfire smoke, produced by the incomplete combustion of lignin, may be adsorbed into ripening grapes resulting in increased VP concentrations at commercial maturity [19]. While found to some extent in their free forms in wines and grapes, the majority of VPs are bound to glycosides containing one or more monosaccharide residue(s) [20,21,22,23]. Enzyme-catalyzed hydrolysis of VP-glycosides during fermentation may result in smoke-tainted wines being produced from berries devoid of objectionable flavors or aromas prior to vinification [24]. VP-glycosides have also been associated with smoky or ashy sensory attributes, although this appears to be the result of released free VP rather than an organoleptic attribute of the VP-glycoside [7,25]. Given the financial impact of vineyard smoke exposure, it is crucial that analytical methods are designed to measure free and bound VPs in grapes and wine to test their potential to yield perceptible smoke-taint.

To date, a variety of analytical methods have been developed by academic and fee-for-service laboratories around the world [4,21,26,27,28,29,30,31,32]. The majority of smoke-taint detection methods rely on gas chromatography-mass spectrometry (GC-MS) or tandem MS (MS/MS) to detect free VPs; these methods are also suitable for glycosidically bound VPs, provided that they have been previously released by acid- or enzymatic-catalyzed hydrolysis [13,30,33]. Existing methods from the peer-reviewed literature for grape and wine sample preparation include liquid–liquid extraction (LLE) [27,30,34], solid phase extraction (SPE) [28,30], and solid phase microextraction (SPME) to isolate the analytes prior to quantitation. The exact conditions used in these methods vary considerably [28,29,30,31]. In addition to the hydrolysis-based GC methods for the indirect quantitation of VP-glycoconjugates, there have also been methods developed for these analytes that employ SPE followed by direct HPLC-MS quantitation [22,26]. These HPLC-MS-based methods are complicated with a potentially large [23] and variable range of VP-glycoconjugates (which are difficult to deduce a priori) and, until recently, a dearth of commercially available analytical standards. Nevertheless, glycoconjugates are important to quantitate because they represent a data set that can inform the risk assessment of a given sample.

Quantitative methods are used to help grape producers, and wineries make informed decisions about the potential risks of fermenting smoke-exposed grapes and bottling/aging the resulting wines. Additionally, these methods may assist in the development of mitigation and/or crop-protection strategies. Since the results of smoke-taint testing can be the deciding factor in multimillion-dollar decisions, it is imperative that the analytical strategies employed provide timely, accurate, and precise data. Herein, the data from an inter-laboratory study are reported, with nine participating laboratories from around the world quantitating VPs and their presumptive glycoconjugates in the same set of ten wine samples. This study had three primary objectives: (1) to function as a formal proficiency testing assessment (per requirements of ISO/EIC 17025 accredited testing facilities); (2) to establish the variance amongst commercial and research laboratories, which will help the wine industry and academicians understand how results from different studies and/or service providers can be compared; and (3) to evaluate correlations between quantitative sensory assessments and the calculated consensus values for VPs (free and glycosidically bound).

## 2. Results and Discussion

### 2.1. Analytical Methods

The analytical methods employed for the quantitation of free (Table 1) and intact (Table S1) VPs in the wines sourced for the inter-laboratory study (Table 2) cover the range of methods developed to date [4,14,21,26,27,28,29,30,32]. Without exception, all participants (denoted by lab IDs to ensure anonymity) used GC-MS or GC-MS/MS for the quantitation of free VPs, with all reported column chemistries falling under DB5 or WAX-type stationary phases. Previous results have demonstrated that those labs using WAX-type column chemistry will be able to chromatographically resolve all three cresols, enabling their quantitation separately; this is not possible on a DB5 phase on a time-scale suitable for fee-for-service testing (*m*-cresol and *p*-cresol will co-elute [30]). Only three out of nine participants employed SPME for sample analysis, while five out of nine used acid-catalyzed hydrolysis to indirectly quantitate bound VPs, and only three out of nine study participants reported values for intact VP-glycosides (Table S1).

This self-reported method information, some of which has been published elsewhere in more detail [1,4,13,26,27,28,29,30,31,32,35], highlights several points of divergence in testing methodologies that are worthy of further discussion within the smoke-taint testing community. In particular, the impact of sample preparation for free VPs (LLE vs. SPME), how cresols are reported (individual vs. summation), LOQ variability, and how bound VPs are quantitated (after release to the free VP vs. intact analysis) warrant comparison beyond the data herein (vide infra). Consensus methods that are codified by organizations such as AOAC International, Codex Alimentarius, or International Organization of Vine and Wine (OIV) are typically used to address such concerns around the impact of different methods on critical analytical metrics. However, given the relatively small market (on a global scale) for smoke-taint testing services, it is likely to fall on smoke-taint testing stakeholders to discuss the need for consensus methods and their subsequent adoption.

### 2.2. Study Accuracy

To assess laboratory accuracy, a model wine (Table S2) was fortified with free VPs: 33 µg/L phenol, 29 µg/L *o*-cresol, 41 µg/L *m*-cresol, 14 µg/L *p*-cresol, 6 µg/L guaiacol, and 9 µg/L 4-methylguaiacol. This approach was used due to a lack of certified reference materials for smoke-taint and the fact that most commercial wines have at least trace amounts of VPs, most notably guaiacol. While not an ISO17034 certified reference material producer, the model wine was prepared in an ISO17025 accredited facility with appropriate metrological traceability and quality management procedures to be suitable for the purposes of this inter-laboratory comparison.The data demonstrated accuracies of 44–136% when all participant results were included (Table 3). However, lab 407 was identified as having a potential systematic error in their analysis, so with that data removed, the reported accuracies were 54–136%. The relative standard deviations (RSD) amongst each VP were 11–29% (again with lab 407 removed). For guaiacol, the classical VP marker associated with smoke-taint, the mean accuracy was 94 ± 11% (SD)—a result that indicates suitable aggregate method accuracy given the size of this study and the low-part per billion (ppb) concentrations being analyzed. Overall, the model wine data suggest a potential negative bias in the accuracy results, but given the study size and the fact that certified reference materials were not being used, the aggregate data for all analytes (except *p*-cresol precision with a 27% RSD) are within acceptable tolerances. It should also be noted that only four out of nine participants included phenol in their analysis, so the data herein are directional rather than absolute for this analyte.

### 2.3. Inter-Laboratory Results

The aggregate consensus values for free VPs (Table 2; see Table S3 for replicates used to calculate consensus values) in the ten wines used in this study demonstrated results that were broadly consistent with the model wine data, as well as with the expected variation in precision as a function of absolute analyte concentration (i.e., a decrease in relative precision at lower concentrations). Of note regarding method precision were *m*-cresol, phenol, and syringol. These analytes showed generally higher SDs than the other free VPs. As noted, however, the number of study participants for phenol was only four, so the expected imprecision for these consensus values would be higher. Syringol has historically been a problematic compound both chromatographically and from a (presumptive) reactivity standpoint (unpublished observations). As such, higher imprecision was anticipated. While all of the cresols displayed aggregate precision that was worse than guaiacol or 4-methylguaiacol, *m*-cresol stands out amongst these structural isomers as having overall higher imprecision within a given wine sample when compared to *o*/*p*-cresol. The source of variability for the cresols is not clear based on the design of this study, but it is of concern given the demonstrated correlation between o-cresol and the perception of ashy flavors and aromas [7]. These data strongly support a conservative approach when comparing free VP data between laboratories, especially when considering cresols. Correlative discussion about the consensus values and perceived smoke-taint is discussed elsewhere (vide infra).

At the core of any inter-laboratory study is the calculation of the *z*-score, which is used as a means to assign a pass/fail designation to each study participant. Such testing programs are required, for instance, as part of ongoing ISO17025 accreditation for a given test method. Due to the relatively small number of study participants, the lack of known variability from previous inter-laboratory studies on smoke-taint and the observed variability in the study results herein, a classical definition of *z*-score (i.e., deviation from the mean normalized to standard deviation) was deemed unsuitable as a means to assess participant performance. In lieu, a modified z-score that utilized the aggregate RPD across all analytes and all participants for the blinded sample duplicates was applied to evaluate participant performance. As part of the inter-laboratory study design, a series of blinded duplicate samples was provided to each participant. These were included to obtain an objective assessment of intra-laboratory precision (Table S4). Given the discussed limitations of the study reported here, the mean relative percent difference (RPD) of the blinded duplicates was also utilized to calculate the modified *z*-scores. Looking only at the free VP RPDs from the blinded sample duplicates (Table S4), the mean values for a given participant ranged from 3 to 23%, with a mean of 11%. Performance criteria for laboratory duplicates will vary, but in general, a value of ±20% can be viewed as acceptable. Given these criteria, seven out of nine participants presented free VP data with acceptable inter-laboratory precision.

The modified *z*-scores presented a wide array of passing and failing results, with trends apparent as a function of participant and analyte (Table 4). Looking broadly at the two most widely tested and cited smoke-taint marker compounds, guaiacol and 4-methylguaiacol, it can be seen that the majority of participants are within study tolerances (green-colored cells < ±2 standard deviations from study mean) for all the wines evaluated. Indeed, only 4-methylguaiacol had hard failures (red-colored cells > ±3 standard deviations) for a handful of participants in different wines, two of which were white wines where the free 4-methylguaiacol concentrations were <1.6 µg/L (wines B and E). The three failures for 4-methylguaiacol in wine I are concerning, however, as this was a red wine with a consensus value of 19.0 ± 3.51 µg/L and, for the failing results, the *z*-scores were > 4 standard deviations away from the study mean.

### 2.4. Bound Forms

Owing to their importance in the overall risk for perceptible smoke-taint, the bound forms of VPs (presumptively VP-glycosides [23,26]) were included in the scope of this study. More specifically, the VPs quantitated in wines that were subjected to acid-catalyzed hydrolysis before extraction are inferred to reflect the sum of both the free VPs (aglycones) and all glycosidically bound analogues, with the difference corresponding to the putative bound fraction. Only five (out of nine) participants reported VP levels following acid-catalyzed hydrolysis, while three (out of nine) reported values for intact VP-glycosides, so the statistical significance of these data was not as sound as that for most of the free VPs. For the results reported post-acid hydrolysis, the consensus values for all analytes evaluated were less precise than the free VPs (Table 2), with typical RSDs of 40–60% and some showing RSDs in excess of 100%. Similar results are further reflected in the blind duplicates that underwent bound VP evaluation (Table S5), with a mean RPD of 20 ± 25%.

Problematically, there were numerous instances (especially notable for wines H and I) where the consensus values for the free VPs exceed those evaluated after acid hydrolysis, reflecting a failure to achieve mass balance, as previously noted by Szeto and colleagues [36]. Note that previous research has indicated that both acid (H_2_SO_4_ vs. HCl) and the hydrolysis vessel (glass vs. PTFE) significantly influence VP recoveries post-hydrolysis [30]. As noted above, a more direct approach involving the quantitation of intact VP-glycosides (Table S6) is complicated by the large diversity of possible analogues and limited sets of standards. Here, between three labs, a total of ten VP-glycosides were quantitated, although only one (guaiacyl-β-D-glucopyranoside) was evaluated in duplicate with a mean inter-laboratory RPD of 76 ± 52%. The development of more accurate and precise methods for bound forms of smoke-taint-associated VPs should be prioritized for several reasons. First, Caffrey and co-workers have demonstrated [23], using an extensive range of putative VP-glycosides, that only about three quarters of VP-glycosides are hydrolyzed by yeast glycosidases during the initial stages of primary fermentation, while Ristic and co-workers have provided evidence indicating that VP-glycosides remain stable during wine aging [1]. However, VP-glycosides may not be the sole source of VPs in wines, as two independent field studies [22,36] have indicated the presence of bound VPs that are not simple glycosides; at the moment it is not known if these forms of bound VPs are stable towards acid-catalyzed hydrolysis (during aging) or enzyme-catalyzed breakdown during fermentation. Further, since the majority of free VPs in wines originate from bound precursors, improved methods for quantitating the latter are imperative for guiding the development of winery-based smoke-taint amelioration techniques.

### 2.5. Comparison of Sensory Profiles of Control and Smoke-Tainted Wines

**Figure 1:**
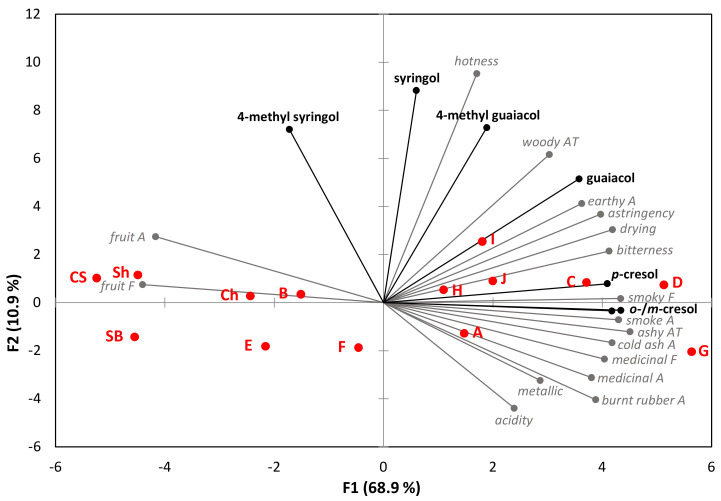
Principal component analysis biplot of free VP concentrations and mean sensory attribute ratings of control (Ch—Chardonnay, SB—Sauvignon Blanc, CS—Cabernet Sauvignon, Sh—Shiraz) and smoke-tainted (A to J) wines. A—aroma attribute; F—flavor attribute; AT—aftertaste.

Significant differences were not only perceived between the sensory profiles of control and smoke-tainted wines but also among the ten smoke-tainted wines (Table S7). As a consequence, PCA of sensory data (overlaid with wine volatile phenol concentrations) gave a biplot with clear separation of wines along the *x*-axis (Figure 1). The first principal component (PC) explained 68.9% of variation and separated wines according to the prominence of fruit aroma and flavor vs. smoke-related attributes. As such, the four control wines, which exhibited prominent fruit characters, were located on the left side of the biplot; the control Sauvignon Blanc, Cabernet Sauvignon, and Shiraz were clustered together, whereas the control Chardonnay, which exhibited less intense fruit characters and smoky, woody notes due to oak maturation, was positioned slightly to their right. Wines C, D, and G exhibited the most intense smoke, cold ash, medicinal and burnt rubber aromas and flavors, and ashy aftertaste, and therefore diminished fruit attributes. As such, these wines were positioned on the far right of the biplot. Wines A, H, I, and J were also positioned on the right side of the biplot, but these wines exhibited moderate fruit intensity, and earthy, medicinal, and/or burnt rubber characters, rather than overt smoke and cold ash notes, albeit all four gave a discernible ashy aftertaste. These wines were therefore clustered closer to the origin than Wines C, D, and G. The remaining wines, Wines B, E, and F, were located to the left of the biplot’s origin. These wines exhibited moderate to high fruit intensity and less apparent smoke-related aromas and flavors, such that there were fewer significant differences between their sensory profiles and those of the control wines. The second PC only explained a further 10.9% of variation and separated wines based on their perceived hotness (Figure 1), while the third PC was driven by acidity and explained a further 7.4% of variation (data not shown).

When the VP concentrations of control and smoke-tainted wines were overlaid onto the PCA biplot of sensory data, the concentration of cresols appeared to most strongly drive the position of wines along the *x*-axis. Cresols were only detected (at 1 µg/L) in two of the control wines, but guaiacol, 4-methylguaiacol, syringol, and 4-methylsyringol were present (at 4–12, 7–11, 16–51, and 26–39 µg/L, respectively) in the control Chardonnay, Cabernet Sauvignon, and Shiraz wines, and even the control Sauvignon Blanc contained 2 µg/L of guaiacol and 9 µg/L of syringol (Table S8), without any suggestion of smoke taint. The presence of VPs in control wines is largely attributable to oak maturation [14], but some VPs are known to occur naturally in selected grape varieties, Shiraz in particular [27,37].

The smoke-tainted wines positioned on the left side of the biplot (Wines B, E, and F) had lower levels of VPs (including cresols) than wines on the right side of the biplot. The wines perceived to be the most heavily tainted by sensory analysis (Wines C, D, and G) had the highest total cresol levels (at ~30–38 µg/L), whereas the moderately smoke-tainted wines (Wines A, H, I, and J) had moderate cresol levels (~10–28 µg/L). Some of the highest guaiacol and syringol levels were detected in Wines H, I, and J, however, these wines were not deemed the most heavily smoke-tainted by sensory analysis. These results suggest the cresols might be better markers for predicting the sensory perception of smoke taint, than the other smoke-derived volatile phenols.

**Figure 2:**
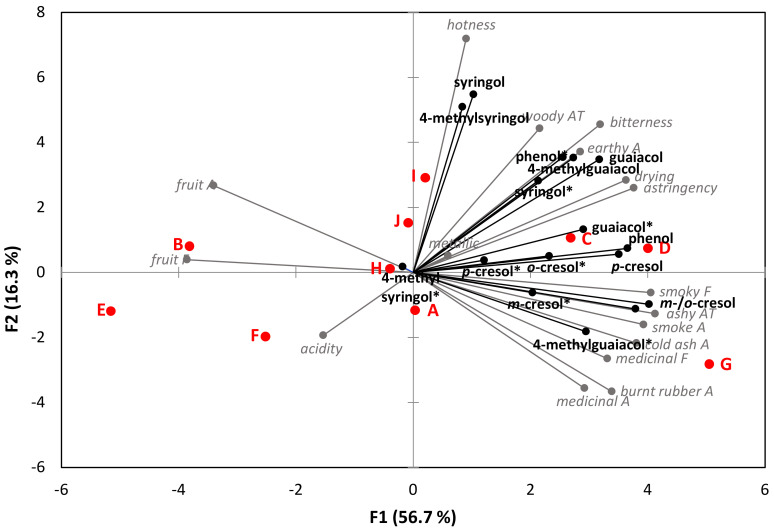
Principal component analysis biplot of free and bound (*) volatile phenol concentrations and mean sensory attribute ratings of smoke-tainted (A–J) wines. A—aroma attribute; F—flavor attribute; AT—aftertaste.

The relative impact of VPs (in free and bound forms) on the sensory perception of smoke taint was further investigated by overlaying the PCA biplot of sensory data for the 10 smoke-tainted wines with their compositional data (Figure 2). The first and second PCs explained 73% of variation, and wines were positioned along the biplot *x*-axis in a similar distribution as before. However, in addition to (free) cresols, phenol, and bound forms of guaiacol, 4-methylguaiacol, and cresols appear to be driving separation of wines. This might reflect the contribution of glycosylated VPs to the perception of smoke taint proposed by Mayr and colleagues [25].

## 3. Materials and Methods

### 3.1. Inter-Laboratory Study Design 

A set of ten commercial wines (Table 2), including seven reds, two whites, and one rosé, were obtained from wineries located in the Okanagan Valley of British Columbia, Canada. The wines were selected based on informal sensory assessments and analytical testing that indicated varying degrees of smoke-taint. Of these ten wines, seven were never marketed due to high levels of smoke-taint perceived after fermentation; three wines were available commercially, albeit identified as smoke-tainted. The commercial wines and a model wine fortified with VPs (Table S2; 33 µg/L phenol, 29 µg/L *o*-cresol, 41 µg/L *m*-cresol, 14 µg/L *p*-cresol, 6 µg/L guaiacol, 9 µg/L 4-methylguaiacol) were sent to participants that were recruited based on their smoke-taint testing capabilities for commercial and/or academic purposes. Additionally, for each participant, a set of four samples were provided as blinded duplicates, with each participant receiving a different set of duplicates. Thus, a total of 15 samples were sent to each participant; the model wine fortified with VPs and 14 commercial wine samples (with four of those being blind duplicates). Participants were instructed to analyze all samples according to their standard procedures for smoke-taint testing (i.e., using their own analytical standards and calibration curves). Method details were provided by each laboratory for free VPs (Table 1) and (if tested) VP-glycosides (Table S1). There was no requirement that participants be able to test a minimum number of VPs, nor was there a requirement that they were able to quantitate bound-VPs and/or VP-glycosides. The final breakdown included nine participants testing free VPs, with all participants testing for guaiacol and 4-methylguaiacol and subsets testing for other VPs, including phenol, *o*-, *m*-, and *p*-cresol, ethylphenols, and syringols. Additionally, five participants tested for bound VPs and three tested for intact VP-glycosides, with the VPs tested for aligning with the free VPs tested by a given participant.

### 3.2. Sensory Analysis of Wines 

Four commercial Australian wines (a 2019 Sauvignon Blanc, a 2018 Chardonnay, a 2018 Cabernet Sauvignon, and a 2018 Shiraz, each approximately AUD 7 per bottle) were purchased from a retail outlet as control wines for sensory analysis. The sensory profiles of control and smoke-tainted wines were determined using the Rate-All-That-Apply (RATA) method [38]. Wines were evaluated by panels comprising 50 staff and students (11–16 males and 34–39 females, aged between 21 and 64 years) from the University of Adelaide, the Australian Wine Research Institute (AWRI), Commonwealth Scientific and Industrial Research Organisation (CSIRO), and South Australian Research and Development Institute(SARDI), during three sensory sessions; white and rosé wines were evaluated in session one, and red wines were evaluated in sessions two and three, such that no more than five wines were evaluated per session. Prior to each evaluation, panelists were familiarized with the RATA procedure and the list of attributes and their definitions (Table S9) adapted from previous studies [36,37]. RATA assessments were conducted in sensory booths at 22–23 °C, with wine aliquots (30 mL) presented monadically, at ambient temperature, in a randomized order, in covered, 4-digit coded 215 mL stemmed International Organization for Standardization (ISO) wine glasses. Panelists rated the intensity of each sensory attribute using seven-point Likert scales (where 1 = ‘extremely low’ and 7 = ‘extremely high’); if attributes were not detected, panelists did not need to give a score (in which case the intensity was rated as 0). Panelists rinsed thoroughly with water between samples; plain crackers were also provided as palate cleansers. Data were acquired with Red Jade software (Redwood Shores, CA, USA). The concentrations of VPs present in control wines were also determined by GC-MS, as described previously [14,26].

### 3.3. Data Analysis

Sensory data were analyzed by two-way analysis of variance (ANOVA) using participants as a random factor and wines as a fixed factor, with Fischer’s LSD post hoc test (*p* ≤ 0.05), to determine significant differences between wines, using XLSTAT (version 2018.1.1, Addinsoft, New York, NY, USA). Mean comparisons were performed by least significant difference (LSD) multiple comparison test at *p* < 0.05. Principal component analysis (PCA) of sensory data was performed using XLSTAT, including with chemical data overlaid.

An assumption of normality was used for all inter-laboratory statistical calculations. Due to the observed disparity of the inter-laboratory results, absence of reported variance when comparing VP results between laboratories, and the number of study participants, the classical version of *z*-scoring (where the denominator is the standard deviation of the study data) was not used. Rather, an approach that utilized the objective variance of the inter-laboratory study blind duplicates was utilized in the calculation of a modified *z*-score (Equation (1)):

(1) $$z=\frac{x_{i}-\bar{x}}{RPD_{dup}\times \bar{x}},$$

where *x_i_* = the calculated concentration for a given analyte, $\bar{x}$ = the consensus (mean) value for a given analyte, and *RPD_dup_* = the aggregate relative percent difference (RPD) across all analytes and all participants for the blinded sample duplicates. Consensus values were calculated for each analyte from the mean of the data supplied by each participating laboratory. For a given data set, the presence of outliers was evaluated using a Grubb’s test (α = 0.05, *n* −2 degrees of freedom (i.e., two-tailed test); Equation (2)):

(2) $$RPD=\frac{\left|x_{1}-x_{2}\right|}{\left(\frac{x_{1}+x_{2}}{2}\right)}\times 100,$$

where *x_i_* = the calculated concentration for a given analyte, $\bar{x}$ = the consensus (mean) value for a given analyte, and s = the standard deviation of the values used to calculate $\bar{x}$. If *g* ≥ g_crit_, then the value was excluded from the determination of a given consensus value. No more than one outlier was excluded from each data set. Intra-laboratory precision was calculated as the relative percent difference (RPD) between blinded duplicates (Equation (3)):

(3) $$g=\frac{max_{1-i}\left(x_{i}-\bar{x}\right)}{s},$$

where *x*_1_ and *x*_2_ = the calculated concentrations submitted for a given set of duplicate samples.

## 4. Conclusions

The interlaboratory comparison reported here demonstrated that for the classically discussed smoke-taint markers (guaiacol and 4-methylguaiacol), laboratory results are broadly comparable. However, when evaluating other volatile phenols, the results reported between laboratories should be compared with caution due to the imprecision observed herein. These findings extend to comparisons of glycosidically bound smoke-taint markers (be they acid-hydrolyzed or intact glycoside analyses). While the study results for these tests were underpowered, when viewed directionally, they suggest caution be used when comparing results between commercial laboratories and amongst the published peer-reviewed manuscripts. Additionally, the sensory assessments reported herein have indicated that cresols should be considered as key marker compounds for predicting the likely perception of smoke-taint. Problematically, however, the concentrations of the cresols as a group exhibited the most inter-laboratory variance of the VPs quantitated in this study; additionally, in some instances, the cresols also exhibited substantially more intra-laboratory variance as assessed by the relative percent differences among blinded sample duplicates.

## Figures and Tables

**Table 1:** Method parameters as provided by study participants.

Lab ID			1	2	3	4	5	6	7	8	9
**free** **VPs**	**processing**	**technique**	LLE ^2^	LLE	LLE	SPME	LLE	SPME	LLE	LLE	SPE
		extraction solvent	pentane: EA (1:1)	pentane: ether (2:1)	hexane: EA (1:1)	nr ^1^	na ^1^	na	pentane: EA (1:1)	nr	SPE
		NaCl added	no	no	yes	nr	no	yes	no	no	no
		sample vol. (mL)	5	5	5	nr	5	10	5	30	3
		conc. factor	2.5	2.5	2.5	nr	2	na	2.5	15	2
	**analysis**	**separation**	GC-MS	GC-MS	GC-MS/MS	GC-MS/MS	GC-MS	GC-MS/MS	GC-MS	GC-MS	GC-MS ^3^
		SPME fiber	na	na	na	PDMS/DVB	PDMS/DVB	nr	na	na	na
		GC column	DB5	nr	DB5	WAX	WAX	nr	WAX	WAX	DB-1701
		inj. volume (µL)	2	1	5	na	na	na	2	1	1
		LOQs (ug/L)	1	1	1	5	na	1 ^4^	0.5	na	3–5
		ISTDs	yes	yes	Yes	yes	yes	yes	yes	yes	yes
**total** **VPs (H^+^)**	**processing**	**extraction solvent**	na	pentane: ether (2:1)	hexane: EA (1:1)	na	na	na	na	nr	SPE ^5^
		salting out	na	no	Yes	na	no	na	na	na	na
		conc. factor	na	2.5	2.5	na	2	na	na	nr	2
		incubation time (h)	na	nr	4	na	1	na	na	na	1
		temperature (°C)	na	100	100	na	100	na	na	na	100
		incubation vessel	na	glass	PTFE	na	glass	na	na	na	glass
		acid; conc.	na	nr	HCl; 1 N	na	H_2_SO_4_; 1N	na	na	na	H_2_SO_4_; 5N
		enzyme(s)	na	na	na	na	na	na	na	na	na

^1^ na—not applicable, i.e., the parameter does not apply to a particular protocol; nr—details were not reported by participant(s). ^2^ The following abbreviations are used: LLE—liquid–liquid extraction; SPME—solid phase micro extraction; SPE—solid phase extraction; EA—ethyl acetate; LOQ—limit of quantitatuion; conc.—concentration; inj.—injection; ISTD—internal standard; PTFE—polytetrafluoroethylene; PDMS/DVB—polydimethylsiloxane/divinyl benzene. ^3^ GC-MS following trimethylsilane derivatization. ^4^ All LOQs 1 μg/L except syringol = 10 μg/L. ^5^ VPs were reported as separate free and bound values after sample preparation.

**Table 2:** Details of the wines used for the inter-laboratory comparison and consensus-free and bound VP results. Replicates used to generate the aggregate values are summarized in Table S3.

Wine ID		A	B	C	D	E	F	G	H	I	J
**vintage**		2018	2018	2018	2018	2018	2018	2019	2015	2105	2018
**varietal ^1^**		R.B.	Ch	Merlot	C.F.	P.B.	Rosé	P.N.	C.F.	R.B.	Syrah
**VP**	**H+ ^2^**	**concentration (μg/L) ^3,4,5^**									
4-MG	−	4.75 (0.73)	1.58 (1.27)	9.26 (1.15)	11.6 (1.29)	1.39 (0.39)	1.99 (0.22)	10.9 (1.46)	11.12(2.05)	19.0 (3.51)	4.11 (1.33)
4-MG	+	7.52 (3.41)	5.93 (2.28)	20.6 (8.62)	12.8 (5.24)	1.39 (0.53)	13.7 (4.35)	16.4 (6.05)	7.49 (3.39)	5 (0) #	6.32 (2.83)
4-MS	−	4.58 (0.79)	nd	8.46 (1.33)	5.22 (1.12)	1.66 (0.39)	nd	4.49 (0.76)	6.25 (0.93)	32.5 (2.87)	5.26 (0.85)
4-MS	+								3 (0) #		
guaiacol	−	19.3 (3.21)	3.11 (0.39)	31.1 (6.24)	42.3 (4.53)	1.98 (0.34)	9.45 (1.13)	34.1 (5.13)	39.3 (5.06)	50.5 (10.3)	24.2 (4.21)
guaiacol	+	46.4 (36.4)	22.1 (10.8)	82.4 (48.8)	98.6 (106)	19.54(24.5)	60.7 (22.7)	47.4 (18.7)	39.5 (22.5)	39.8 (36.3)	64.2 (38.5)
*m*-cresol	−	7.17 (2.99)	1.96 (1.22)	11.1 (6.41)	13.7 (6.82)	2.14 (1.22)	3.69 (1.85)	16.9 (6.90)	13.3 (4.46)	8.26 (4.09)	4.13 (2.49)
*m*-cresol	+	23.5 (34.5)	17.8 (22.6)	49.8 (68.1)	40.3 (63.4)	15.7 (26.7)	34.8 (46.5)	24.1 (26.9)	15.2 (21.9)	13.0 (17.4)	20.8 (29.1)
*o*-cresol	−	5.86 (1.54)	1.02 (0.14)	8.02 (2.33)	9.86 (2.97)	1.17 (0.41)	3.02 (0.75)	11.3 (3.11)	7.53 (2.52)	4.85 (1.92)	3.70 (1.51)
*o*-cresol	+	34.4 (51.0)	9.15 (7.24)	33.8 (48.0)	95.3 (151)	5.00 (1.41)	19.7 (22.8)	14.8 (12.0)	12.7 (13.3)	3 (0) #	20.0 (30.2)
*p*-cresol	−	6.31 (1.84)	6.00 (4.36)	10.7 (2.27)	13.6 (2.41)	2.15 (1.30)	3.79 (1.88)	10.1 (1.98)	7.09 (1.92)	6.46 (1.72)	3.49 (1.41)
*p*-cresol	+	9.33 (7.23)	8.00 (5.29)	19.0 (14.8)	9.00 (6.56)	5.00 (3.46)	14.0 (10.2)	8.33 (6.66)	7.33 (6.03)	6.50 (0.71)	9.67 (6.81)
phenol	−	28.5 (3.72)	7.39 (4.28)	36.3 (11.9)	52.5 (15.3)	8.06 (1.07)	13.5 (1.86)	48.6 (5.73)	47.2 (2.57)	41.1 (9.55)	18.2 (2.04)
phenol	+	236 (203)	97.0 (59.4)	337 (291)	397 (404)	84.5 (88.4)	142 (113)	137 (87.0)	115 (83.4)	202 (150)	335 (211)
syringol	−	20.5 (3.88)	1.08 (0.70)	28.8 (4.29)	22.5 (8.22)	1.60 (0.42)	3.84 (3.20)	19.2 (4.81)	53.1 (7.20)	113 (18.4)	49.0 (7.58)
syringol	+	11.0 (3.61)	6.81 (5.63)	19.0 (11.5)	18.0 (11.6)	8.50 (10.6)	4.38 (2.56)	19.0 (9.17)	31.5 (13.7)	24.7 (10.5)	12.3 (7.80)

^1^ The following abbreviations are used for wine: R.B.—Red Blend; Ch—Chardonnay; C.F.—Cabernet Franc; P.B.—Pinot Blanc; P.N.—Pinot Noir. For volatile phenols (VPs), 4-MG and 4-MS refer to 4-methylguaiacol and 4-methylsyringol, respectively. ^2^ H^+^ indicates samples that have (+) or have not (−) been subjected to acid hydrolysis before VP extraction and quantitation. The samples indicated (+), then, are the sum of both free VPs (−) and their glycosidically bound (or other) analogues. ^3^ All data are reported as mean concentrations with standard deviations indicated in parentheses. ^4^ nd = not detected; where VPs were not quantitated, cells are left empty. ^5^ # is used to indicate when two results were reported with only one significant figure at the same nominal value.

**Table 3:** Model wine accuracy (%) for free VPs as provided by study participants ^1,2,3^.

Lab ID	phenol	*o*-cresol	*m*-cresol	*p*-cresol	guaiacol	4-MG
678	121	100	88	107	100	122
675		79	93	114	100	78
314		86	117	136	83	67
782	93	96	95	95	113	96
428		64	64	54	95	61
407	44	59	52	- ^(4)^	48	51
703	109	91	84	- ^(4)^	84	98
660		87	84	77	95	78
101		79	80	79	83	78
**mean**	108	85	88	94	94	85
**SD**	14	11	15	27	11	20

^1^ Lab IDs do not correlate to those in Table 1 to ensure participant anonymity. ^2^ Where no value is provided, the given analyte was not assessed. ^3^ Fortified amounts were 33 µg/L phenol, 29 µg/L *o*-cresol, 41 µg/L *m*-cresol, 14 µg/L *p*-cresol, 6 µg/L guaiacol, 9 µg/L 4-methylguaiacol (4-MG). ^(4)^ *m*/*p*-Cresol co-elute; accuracy reported based on the sum of the fortified amounts for each analyte.

**Table 4:** Summary of inter-laboratory comparison for free VPs using modified *z*-scores ^1,2^.

Lab ID		A	B	C	D	E	F	G	H	I	J	A	B	C	D	E	F	G	H	I	J	Lab ID	
**678**	**phenol**	0.8	6.7	3.3	−1.6		1.6	0.1	−0.6	−1.1	−1.7	2.2		1.4	1.2		1.4	2.0	1.7	1.8	1.3	**guaiacol**	**678**
**675**												−0.6	−0.3	−0.6	−0.5	0.1	−0.4	−1.1	−0.7	−0.1	−1.7		**675**
**314**												−1.0	−0.3	2.2	0.8	0.1	−0.4	−1.1	0.1	1.0	0.6		**314**
**782**		0.2	−0.9	−0.1	−1.3	0.9	0.2	0.0	−0.1	1.0	−1.0	0.8	1.4	0.7		2.3	1.9	0.9	0.7	0.6			**782**
**428**												−1.2	−0.4	−1.4	−0.9	−2.7	−0.5	−1.3	−1.6	−2.9	−1.7		**428**
**407**		−1.7	0.0	−3.7	3.8	0.4	−0.7	−1.3	0.2	−0.6	2.9	−0.5	1.6	−2.7		0.9	0.0	−0.3	1.3	−0.2	2.8		**407**
**703**		0.7	−5.7	0.5	−0.9	−1.3	−1.1	1.2	0.5	0.6	−0.2	2.5		2.4	1.0		0.2	2.0	0.3	1.9	1.5		**703**
**660**												−1.1	−1.6	−0.8	−0.9	−0.8	−0.8	−0.5	−0.9	−0.9	−1.4		**660**
**101**												−1.0	−0.3	−1.2	−0.7	0.1	−1.4	−0.8	−1.0	−1.2	−1.5		**101**
**678**	***o*-cresol**	3.2		2.2	2.8		2.9	4.5	5.2	3.1	3.9		7.9							4.1	2.8	**4-methylguaiacol**	**678**
**675**		−1.3		−1.1	−2.6		−0.1	−1.0	−0.6	−1.7	−1.5	0.5		−0.2	0.3	3.9	0.1	−0.7	−0.1	−0.2	−1.4		**675**
**314**		3.2		4.4	3.7	6.2	2.9	1.4	1.7	5.5	5.7	−1.4		0.7	−1.2	−2.5	0.1	−1.5	−1.7	−0.2	−0.4		**314**
**782**		1.6	0.7	0.4	2.1	−0.6	1.7	1.2	1.1	1.7	1.7	1.4		1.5	1.7	0.7	0.9	1.3	1.0	−0.4	0.4		**782**
**428**		−1.6		−1.9	−0.2		−3.3	−1.3	−1.3	−4.0	−2.1	−1.5		−2.3	−1.1		−1.4	−1.6	−2.9	−4.6	−2.4		**428**
**407**		0.6	−0.5	2.4	−4.8	−1.0	−2.7	−0.1	0.3	2.1	−3.6	2.1	−2.0	0.1	0.3	2.6	1.7	0.7	2.2	1.3	−0.6		**407**
**703**		−3.6	1.6	−3.7	−0.9	−1.0	−0.6	−4.6	−5.9	−5.8	−5.1	0.5		0.3	0.8	−1.3	−0.6	1.4	1.0	4.1	0.5		**703**
**660**		−0.8	−1.7	−1.5	−0.2	−2.3	−0.6	0.1	0.1	0.8	0.7	−0.2	−5.8	0.2	−0.4	−0.9	−0.8	0.2	0.5	−1.6	2.0		**660**
**101**		−1.3	−0.2	−1.1	0.1	−1.3	−0.1	−0.2	−0.6	−1.7	0.3	−1.4		−0.2	−0.5	−2.5	0.1	0.1	−0.1	−2.4	−0.9		**101**
**678**	***m*-cresol**	−1.4		−4.1	−3.7		−1.6	−2.6	−2.9	−0.3	−3.5	2.4		1.9	0.6	2.2	−1.9	1.3	1.1	1.8	1.3	**syringol**	**678**
**675**		−1.4	0.2	−2.5	−3.7	−0.6	0.8	−2.0	−0.9	−2.4	−2.4	−0.2		0.1	−2.9	2.2	−1.9	−0.5	−0.2	−0.2	−1.1		**675**
**314**				10	4.1			6.3	3.7														**314**
**782**		−0.9		−2.3	−1.6	−2.6	−2.1	−1.4	−0.7	−2.0	0.4	1.6		1.1				3.9	1.1	1.3	1.4		**782**
**428**		−2.7		−3.6	−3.2		−3.7	−3.5	−2.1	−4.1	−2.5	−0.8	4.0	−1.3	−1.1	−0.4	−2.5	−1.2	−1.4	−1.6	−0.9		**428**
**407**		6.9	7.3	5.5	7.8	8.5	8.4	5.2	5.5	11	7.7												**407**
**703**		4.7	−1.7	3.2	5.0	3.2	4.8	2.0	1.6	4.9	5.8				6.1		15		1.3		1.8		**703**
**660**		−2.4	−5.8	−3.1	−2.4	−3.8	−2.5	−2.0	−2.0	−2.6	−2.0	−1.1	−4.0	−0.3	−0.8	−0.7	−4.2	−1.5	−0.7	−0.1	−0.6		**660**
**101**		−2.7		−3.3	−2.4	−4.7	−4.0	−2.0	−2.2	−4.5	−3.5	−1.9		−1.5	−1.8	−3.3	−4.2	−1.9	−1.3	−1.3	−1.7		**101**
**678**	***p*-cresol**	3.8	−4.4	1.9	1.6	3.5	2.8	2.5	3.6	3.8	3.5											**4-methylsyringol**	**678**
**675**		2.4	−2.9	2.7	2.2	7.6	7.5	1.7	2.4	3.8	2.1	0.8		0.6	−2.1	1.8		1.0	1.1	1.2	−0.1		**675**
**314**			7.3																				**314**
**782**		0.0		−0.6	−0.1	−2.3	−1.4	−0.6	−1.0	0.3	−0.5										0.4		**782**
**428**		−2.8		−0.7	−0.8		−4.4	−0.4	−1.5	−5.6	−2.7	0.7		−0.6	1.9	−1.3		0.2	−0.5	−1.1	0.6		**428**
**407**		−	−	−	−	−	−	−	−	−	−												**407**
**703**		−	−	−	−	−	−	−	−	−	−												**703**
**660**		−1.5		−1.9	−1.2	−4.1	−2.7	−1.4	−2.2	−1.0	−1.7	−2.3		−1.6	−1.1	−2.2		−2.2	−1.7	−1.4	−1.3		**660**
**101**		−1.8		−1.4	−1.7	−4.7	−1.8	−1.8	−1.4	−1.2	−0.6	0.8		1.6	1.3	1.8		1.0	1.1	1.2	0.4		**101**

^1^ Lab IDs do not correlate to those in Table 2 but are consistent with those in Table 3. ^2^ Green shading: < ±2 standard deviations from the mean consensus values (pass); yellow: ±3 > *x* > ±2 standard deviations from the mean (warning); red: > ±3 standard deviations from the mean (fail); grey: analyte either not evaluated or was a statistically verified outlier; white: below participant reporting limit. Participants 407 and 703 reported *m*/*p*-cresol as a summation due to chromatographic overlap.

## Data Availability

All data are included in the article and/or Supplementary Materials.

## Supplementary Materials

The following supporting information can be downloaded at: https://www.mdpi.com/article/10.3390/molecules27154892/s1, Table S1: Method parameters for intact VP-glycosides as provided by study participants; Table S2: Model wine composition; Table S3: Details of the wines used for the inter-laboratory comparison and sample replicates used to calculate the consensus values; Table S4: Relative percent difference (RPD) for free VPs from blinded sample duplicates; Table S5: Relative percent difference (RPD) for total VPs from blinded sample duplicates; Table S6: Details of the wines used for the inter-laboratory comparison and intact VP-glycoside results; Table S7: Mean intensity ratings for sensory attributes of control and smoke-tainted wines; Table S8: Concentrations of volatile phenols (µg/L) in control wines used for sensory analyses; Table S9: Aroma and palate attributes used in sensory analysis.

## Sample Availability

Samples of the compounds are not available from the authors.
