# Supplementary material for: Correlating Sensory Assessment of Smoke-Tainted Wines with Inter-Laboratory Study Consensus Values for Volatile Phenols
Source: Molecules. 2022 Jul 30;27(15):4892. doi: 10.3390/molecules27154892 (PMC9369848; doi:10.3390/molecules27154892)
Supplement: Supplementary file 1 [file molecules-27-04892-s001.zip › molecules-1809630-supplementary.pdf]

Supplementary Materials

Correlating Sensory Assessment of Smoke-Tainted Wines with Inter-Laboratory Study Consensus Values for Volatile Phenols

James, W. Favell[1,†], Kerry Wilkinson[2,3,†], Ieva Zigg[1], Renata Ristic[2,3], Carolyn J. Puglisi[2], Eric Wilkes[4], Randell Taylor[4], Duane Kelly[5], Greg Howell[5], Marianne McKay[6], Lucky Mokwena[6], Tim Plozza[7], Pei Zhang[7], AnhDuyen Bui[7], Ian Porter[8], Orrin Frederick[9], Jasha Karasek[9], Colleen Szeto[2,3,10], Bruce Pan[10], Steve Tallman[10], Beth Anne McClure[10], Hui Feng[10], Eric Hervé[11], Anita Oberholster[12], Wesley F. Zandberg[1,*], and Matthew Noestheden[1,13,*]

[1] The University of British Columbia, Kelowna, British Columbia, Canada; jfavell@ualberta.ca (J.F.); ieva.zigg@ubc.ca (I.Z.);
[2] The University of Adelaide, Waite Research Institute, Glen Osmond, South Australia, Australia; kerry.wilkinson@adelaide.edu.au (K.W.); renata.ristic@adelaide.edu.au (R.R.); carolyn.puglisi@adelaide.edu.au (C.P.); colleen.szeto@adelaide.edu.au (C.S.);
[3] The Australian Research Council Training Centre for Innovative Wine Production, Glen Osmond, South Australia, Australia;
[4] The Australian Wine Research Institute, P.O. Box 197, Glen Osmond, South Australia, Australia; eric.wilkes@awri.com.au (E.W.); randell.taylor@awri.com.au (R.T.);
[5] Vintessential Laboratories, Dromana, Victoria, Australia; duanekelly@hotmail.com (D.K.); greg@ferment.com.au (G.H.)
[6] Stellenbosch University, Central Analytical Facilities, Stellenbosch, South Africa; marianne@sun.ac.za (M.M); mokwenal@sun.ac.za (L.M.);
[7] Agriculture Victoria, Bundoora, Victoria, Australia; tim.plozza@agriculture.vic.gov.au (T.P.); p.zhang@latrobe.edu.au (P.Z.); anhduyen.bui@agriculture.vic.gov.au (A.B.);
[8] LaTrobe University, Bundoora, Victoria, Australia; i.porter@latrobe.edu.au (I.P.);
[9] Vinquiry, Windsor, California, USA; orrin.frederick@enartis.com (E.F.); jasha.karasek@enartis.com (J.K.);
[10] E. & J. Gallo Wineries, Modesto, California, USA; bruce.pan@ejgallo.com (B.P.); steve.tallman@ejgallo.com (S.T.); BethAnne.McClure@ejgallo.com (B.M); hui.feng@ejgallo.com (H.F);
[11] ETS Laboratories, St Helena, California, USA; eherve@etslabs.com (E.H.);
[12] University of California Davis, Davis, California, USA; aoberholster@ucdavis.edu (A.O.);
[13] Supra Research and Development, Kelowna, British Columbia, Canada
* Correspondence: wesley.zandberg@ubc.ca (W.Z.); info@suprarnd.ca (M.N.)
† These authors contributed equally to this work.

Table S1. Method parameters for intact VP-glycosides as provided by study participants[1,2].

lab ID			675	703	428
intact VP-glycosides	processing	technique	direct sample injection	SPE	nr
		SPE phase	na	polymeric	nr
		salting out	na	no	nr
		sample amount	1 mL	1 mL	nr
		conc. factor	1	1	nr
	analysis	technique	LC-MS/MS	LC-QToF	nr
		LC column	Synergi Hydro-RP 150 x 2.1 mm, 4 µm	Luna Omega C18, 50 x 2.1 mm, 2.6 µm	nr
		inj. volume (µL)	10	20	nr
		LOQ (µg/L)	1	nr	nr
		LOD (µg/L)	nr	2-50	nr
		ISTD-corrected	yes	yes	nr

[1] The following abbreviations are used: SPE = solid phase extraction; na = not applicable; nr = not reported; LOQ and LOD = limits of quantitation and detection, respectively. LC = liquid chromatograph; QToF = quadrupole time-of-flight mass spectrometry; and ISTD = internal standard. [2] Lab IDs do not correspond to those listed in Table 1, but are consistent with those listed in Tables 3 and 4; labs not listed did not test for intact VP-glycosides.

Table S2. Model wine composition.

component	composition (g/L)
ethanol	12 % (v/v)
tartaric acid	5.0
malic acid	3.5
acetic acid	0.60
glucose	2.0
fructose	2.0
NaCl	0.20
KH_2PO_4	2.0
$MgSO_4$	0.40

Table S3. Details of the wines used for the inter-laboratory comparison and sample replicates used to calculate the consensus values.

**wine ID**		**A**	**B**	**C**	**D**	**E**	**F**	**G**	**H**	**I**	**J**
**vintage**		2018	2018	2018	2018	2018	2018	2019	2015	2015	2018
**varietal**		R.B.[1]	Ch	Merlot	C.F.	P.B.	Rosé	P.N.	C.F.	R.B.	Syrah
**VP**	**H+** [2]	**number of replicates**									
**4-MG**	-	8	3	8	8	7	8	8	8	9	9
	+	5	5	5	5	3	5	5	5	2	5
**4-MS**	-	4		4	4	4		4	4	5	4
	+		1	1	1		1	1	2	1	1
**guaiacol**	-	9	7	9	7	7	9	9	9	8	9
	+	5	5	5	5	5	5	5	5	4	5
***m*-cresol**	-	8	4	9	9	6	8	9	9	8	8
	+	4	4	4	4	4	4	4	4	3	4
***o*-cresol**	-	9	5	9	9	6	9	9	9	9	9
	+	3	4	4	3	2	4	3	3	2	4
***p*-cresol**	-	6	3	6	6	5	6	6	6	6	6
	+	3	3	3	3	3	3	3	3	2	3
**phenol**	-	4	4	4	4	3	4	4	4	4	4
	+	2	2	2	2	2	2	2	2	2	2
**syringol**	-	6	2	6	6	5	6	6	7	7	6
	+	3	3	3	4	2	4	3	4	3	4

[1] The following abbreviations are used for wine: R.B. = Red Blend, Ch = Chardonnay, C.F. = Cabernet Franc, P.B. = Pinot Blanc, and P.N. = Pinot Noir. For volatile phenols (VPs) 4-MG and 4-MS refer to 4-methylguaiacol and 4-methylsyringol, respectively. [2] H^+ indicates samples that have (+) or have not (-) been subjected to acid hydrolysis before VP extraction and quantitation. The samples indicated (+), then, are the sum of both free VPs (-) and their glycosidically-bound (or other) analogues.

Table S4. Relative percent difference (RPD) for free VPs from blinded sample duplicates[1,2].

lab ID	wine ID	relative percent difference (%; n = 2)							
		phenol	*o*-cresol	*m*-cresol	*p*-cresol	guaiacol	4-MG	syringol	4-MS
678	A	34	0	15	0	9	0	12	12
678	D	12	7	0	6	0	6	4	10
678	G	2	13	18	0	5	0	0	12
678	I	0	13	18	12	7	4	4	4
675	B	-	-	67	67	0	-	-	-
675	D	-	15	13	16	7	0	7	0
675	F	-	0	29	15	0	0	0	-
675	I	-	0	15	13	2	0	8	3
314	A	-	13	75	58	11	0	-	-
314	B	-	40	22	24	0	-	-	-
314	G	-	17	52	47	3	12	-	-
314	J	-	0	6	19	0	29	-	-
782	C	2	4	2	2	0	0	13	-
782	D	6	1	1	1	-	0	65	-
782	F	5	0	0	0	8	5	-	-
782	H	2	2	0	0	2	3	8	-
428	B	-	-	-	-	11	-	6	-
428	F	-	20	10	26	16	19	28	-
428	H	-	2	3	1	4	8	8	30
428	J	-	41	20	87	44	46	27	38
407	A	9	3	16	-	5	0	-	-
407	C	17	32	4	-	18	0	-	-
407	H	32	24	0	-	26	9	-	-
407	J	12	9	4	-	4	3	-	-
703	A	1	58	2	-	5	2	2	-
703	E	17	12	2	-	65	38	48	-
703	H	3	21	3	-	16	3	28	-
703	I	12	27	13	-	9	11	2	-
660	B	-	10	7	-	4	2	11	-
660	E	-	0	6	5	0	1	7	1
660	G	-	2	2	1	2	2	3	3
660	I	-	2	5	1	2	3	3	5
101	C	-	0	0	12	0	0	0	11
101	E	-	0	0	0	0	0	0	0
101	G	-	0	8	0	0	0	0	0
101	J	-	0	40	0	5	0	2	0

[1] 4-MG = 4-methylguaiacol; 4-MS = 4-methylsyringol. [2] Lab IDs do not correlate to those listed in Table 1, but are consistent with Tables 3 and 4. Only single participant data was available for 4-vinylguaiacol, eugenol, 4-ethylphenol and 4-ethylguaiacol so they were not included in this analysis.

Table S5. Relative percent difference (RPD) for total VPs from blinded sample duplicates[1,2].

		relative percent difference (%; *n* = 2)							
**lab ID[(3)]**	**wine ID**	**phenol**	***o*-cresol**	***m*-cresol**	***p*-cresol**	**guaiacol**	**4-MG**	**syringol**	**4-MS**
678	A	1	21	7	10	1	0	5	12
678	D	15	0	5	7	3	6	3	10
678	G	14	16	11	4	2	3	0	12
678	I	13	22	18	0	5	3	3	4
675	B	-	29	0	18	13	0	60	-
675	D	-	22	24	7	1	13	12	-
675	F	-	18	6	9	5	0	35	-
675	I	-	29	22	13	18	0	16	-
314	A	-	-	-	-	25	0	-	-
314	B	-	-	-	-	0	40	-	-
314	G	-	-	-	-	11	0	-	-
314	J	-	-	-	-	9	18	-	-
703	A	71	54	49	-	92	36	-	-
703	E	19	-	31	-	76	-	-	-
703	H	58	110	51	-	72	55	121	-
703	I	16	-	2	-	12	-	-	-
101	C	-	-	0	40	7	13	18	67
101	E	-	-	0	0	0		0	
101	G	-	-	40	0	0	0	17	0
101	J	-	0	67	0	5	0	8	67

[1] 4-MG = 4-methylguaiacol; 4-MS = 4-methylsyringol. [2] Lab IDs do not correlate to those listed in Table 1, but are consistent with Tables 3 and 4. Only single participant data was available for 4-vinylguaiacol, eugenol, 4-ethylphenol and 4-ethylguaiacol so they were not included in this analysis. [3] Participant 678 data calculated from the sum of reported free and bound VPs.

Table S6. Details of the wines used for the inter-laboratory comparison and intact VP-glycoside results[1, 2].

wine ID	A	B	C	D	E	F	G	H	I	J
**vintage**	2018	2018	2018	2018	2018	2018	2019	2015	2015	2018
**varietal**	R.B.[1]	Ch	Merlot	C.F.	P.B.	Rosé	P.N.	C.F.	R.B.	Syrah
**VP-glycoside**					**concentration (μg/L)**					
**675**										
4MS-gentiobioside	nd	nd	nd	nd	nd	nd	nd	nd	nd	nd
phenol-rutinoside	15	10	17	13	3	9	21	7	5	11
cresol-rutinoside	22	14	35	24	5	20	30	17	9	18
guaiacol-rutinoside	27	7	28	26	2	15	36	7	11	21
4MG-rutinoside	18	7	37	27	2	21	43	9	6	13
syringol-gentiobioside	5	2	7	5	nd	5	8	9	13	3
**703**										
phenol-glucoside	166	22	381	160	nd	273	105	18	nd	88
p-cresol-glucoside	10	nd	25	nd	nd	73	nd	nd	48	7
guaiacol-gentiobioside	10	37	35	28	19	38	25	8	19	13
guaiacol-glucoside	19	7	37	20	nd	30	13	10	nd	23
**428**										
guaiacol-glucoside	38	14	84	36	4	67	50	15	nd	45

[1] The following abbreviations are used for wine: R.B. = Red Blend, Ch = Chardonnay, C.F. = Cabernet Franc, P.B. = Pinot Blanc, and P.N. = Pinot Noir. For volatile phenols (VPs) 4-MG and 4-MS refer to 4-methylguaiacol and 4-methylsyringol, respectively. nd = not detected. [2] Lab IDs do not correlate to those listed in Table 1, but are consistent with Tables 3 and 4.

Table S7. Mean intensity ratings for sensory attributes of control and smoke-tainted wines[1].

	SB	Ch	CS	Sh	A	B	C	D	E	F	G	H	I	J	*P*
fruit A	4.40ab	3.80cde	4.55a	4.46a	2.96fgh	3.66cde	2.96fgh	2.78gh	4.14abc	3.26efg	2.63h	3.80cde	3.88bcd	3.35def	*<0.0001*
smoke A	1.36h	2.08defgh	1.43gh	1.60fgh	2.46bcde	1.68fgh	2.90bc	3.10ab	1.92efgh	2.18cdef	3.82a	2.66bcde	2.73bcd	2.14defg	*<0.0001*
cold ash A	1.30de	1.56de	1.37de	1.28de	2.00bc	1.22e	2.38bc	2.67b	1.60de	1.82cde	3.47a	2.36bc	1.92cde	2.35bc	*<0.0001*
earthy A	1.72e	2.02de	2.06cde	1.92e	2.66abcd	2.34bcde	2.74abc	3.31a	1.74e	2.24bcde	2.41bcde	2.02de	2.73abc	2.82ab	*<0.0001*
medicinal A	1.48de	1.56de	0.96e	1.88bcd	2.62a	1.80cd	2.78a	2.35abc	1.84bcd	2.36abc	3.02a	2.62a	1.71cd	2.53ab	*<0.0001*
burnt rubber A	1.24cde	1.62bcd	0.96de	0.90e	2.28ab	1.16cde	1.78bc	2.20ab	1.46cde	1.46cde	2.59a	1.72bc	1.51cde	1.47cde	*<0.0001*
fruit F	4.24ab	3.74cd	4.37ab	4.42a	3.18efg	3.48def	2.86ghi	2.61hi	3.84bcd	3.30defg	2.51i	3.68de	3.08fgh	3.33defg	*<0.0001*
smoky F	1.42e	1.74de	1.41e	1.44e	2.42cd	1.70de	3.20b	3.37ab	1.42e	2.32cd	4.06a	2.92bc	2.92bc	2.29cd	*<0.0001*
medicinal F	1.36efg	1.26fg	0.78g	1.48def	1.76cdef	1.94cdef	2.12bcd	2.69ab	1.62cdef	2.06bcde	2.94a	2.30abc	1.76cdef	1.92cdef	*<0.0001*
ashy AT	1.32ef	1.74def	1.10f	1.06f	2.54bc	2.04cde	3.28ab	3.57a	1.72def	2.32cd	3.88a	2.62bc	2.41cd	2.45cd	*<0.0001*
woody AT	1.80g	2.50bcdefg	2.31cdefg	2.08efg	2.28cdefg	2.64bcdef	2.64bcdef	2.80abcd	2.14defg	1.94fg	2.98abc	3.08ab	3.41a	2.71abcde	*<0.0001*
metallic	1.70abc	1.40bcd	1.12cd	0.98d	2.06ab	1.70abc	1.90ab	1.82ab	1.96ab	1.50bcd	1.86ab	1.52bcd	1.65abc	2.29a	*0.01*
acidity	3.48cdef	3.44def	3.20ef	3.08f	3.54bcdef	3.78abcd	3.66bcde	3.98abc	4.22a	4.02ab	3.80abcd	3.60bcdef	3.69abcde	3.92abcd	*0.001*
hotness	2.62c	3.00abc	2.82abc	3.02abc	2.76abc	3.06abc	3.22ab	3.22ab	2.74abc	2.74abc	2.67abc	3.12abc	3.20ab	3.29a	*ns*
bitterness	2.30def	2.60cde	1.86f	2.12ef	2.72bcde	2.84abcd	3.46a	3.31ab	2.50cde	2.64cde	3.00abc	2.94abc	3.12abc	3.10abc	*<0.0001*
drying	2.56h	2.78gh	2.65gh	3.00gh	3.94de	2.78gh	4.54bc	5.12a	3.16fg	3.18fg	4.33bcd	3.64ef	4.69ab	4.08cde	*<0.0001*
astringency	2.24f	2.42ef	2.90de	2.92de	3.64bc	2.54ef	4.24a	4.16ab	2.70def	2.74def	4.12ab	3.26cd	4.16ab	3.88ab	*<0.0001*

[1] Values are means of intensity ratings given by 50 panelists. Different letters within rows indicate statistical significance ($P = 0.05$, one-way ANOVA); ns = not significant. Wines labeled A through J are defined in Table S3 above; SB, Ch, CS, and Sh were control wines (Australian) and correspond to Sauvignon Blanc, Chardonnay, Cabernet Sauvignon, and Shiraz, respectively.

Table S8. Concentrations of volatile phenols (μg/L) in control wines used for sensory analyses[1].

Sample	guaiacol	4-MG	*m*-cresol	*o*-cresol	*p*-cresol	syringol	4-MS
Sauvignon Blanc	2	nd	nd	nd	nd	9	nd
Chardonnay	4	11	nd	1	nd	16	34
Cabernet Sauvignon	9	10	nd	nd	1	51	39
Shiraz	12	7	nd	nd	nd	42	26

[1] nd = not detected; 4-MG = 4-methylguaiacol; 4-MS = 4-methysryingol.

Table S9. Aroma and palate attributes used in sensory analysis.

attribute	definition
aroma	
fruit	Intensity of the overall fruit aroma
smoke	Perception of any type of smoke aroma, including smoked meat/bacon, toasty, charry, cigar-box, estery
cold ash	Burnt aroma associate with ashes, including ashtray, tarry, campfire
earthy	Any aroma associated with musty, dusty, wet wood, barnyard, mushroom-like, dank, moldy, stagnant, stale
medicinal	Aromatic characteristic of Band-aids, disinfectant-like, including cleaning products, solvents, chemicals
burnt rubber	Perception of burnt rubber-like aromas
palate	
fruit	Intensity of the overall fruit flavor
smoky	Smoke flavor, including bacon and smoked meat
ashy aftertaste	Ashtray residue perceived in the mouth after expectorating, including coal ash, ashtray, tarry, acrid, campfire
woody aftertaste	Woody residue, including wood, oak, pencil shavings
medicinal	Medicinal flavor, including Band-aids, disinfectant, cleaning products, solvents
metallic	The ‘tinny’ flavor associated with metals
acidity	Intensity of sour/acid taste
hotness	Intensity of warmth/heat due to ethanol
bitter	Intensity of bitter taste, bitter aftertaste
drying aftertaste	Intensity of drying sensation perceived after expectorating
astringency	Intensity of puckering mouthfeel due to tannins
